# Variations in the *TAS2R38* gene among college students in Hubei

**DOI:** 10.1186/s41065-022-00260-x

**Published:** 2022-12-19

**Authors:** Xiaojun Wang, Lin Wang, Mengwei Xia, Feng Teng, Xuejiao Chen, Rufeng Huang, Jiahao Zhou, Juan Xiao, Lihong Zhai

**Affiliations:** 1 School of Basic Medicine, Hubei University of Arts and Science, Xiangyang, 441053 China; 2 Xiangyang Stomatological Hospital, Affiliated Stomatological Hospital of Hubei University of Arts and Science, 441003 Xiangyang, China

**Keywords:** Phenylthiourea, Bitter taste receptor, *TAS2R38*, Polymorphism

## Abstract

**Background:**

The bitter taste receptor gene *TAS2R38* is a member of the human *TAS2R* gene family. Polymorphisms in *TAS2R38* affect the ability to taste the bitterness of phenylthiourea (PTC) compounds, thus affecting an individual’s food preference and health status.

**Methods:**

We investigated polymorphisms in the *TAS2R38* gene and the sensitivity to PTC bitterness among healthy Chinese college students in Hubei province. The association of *TAS2R38* polymorphisms and PTC sensitivity with body mass index (BMI), food preference, and health status was also analyzed. A total of 320 healthy college students were enrolled (male: 133, female: 187; aged 18–23 years). The threshold value method was used to measure the perception of PTC bitterness, and a questionnaire was used to analyze dietary preferences and health status. Polymerase chain reaction (PCR) was used to analyze polymorphisms at three common *TAS2R38* loci (rs713598, rs1726866, and rs10246939).

**Results:**

In our study population, 65.00% of individuals had medium sensitivity to the bitterness of PTC; in contrast, 20.94% were highly sensitive to PTC bitterness, and 14.06% were not sensitive. For the *TAS2R38* gene, the PAV/PAV and PAV/AAI diplotypes were the most common (42.19% and 40.63%, respectively), followed by the homozygous AVI/AVI (8.75%) and PAV/AVI (5.00%) diplotypes.

**Conclusion:**

There was a significant correlation between the sensitivity to PTC bitterness and sex, but there was no correlation between the common diplotypes of *TAS2R38* and gender. Polymorphisms in the *TAS2R38* gene were associated with the preference for tea, but not with one’s native place, BMI, health status, or other dietary preferences. There was no significant correlation between the perception of PTC bitterness and one’s native place, BMI, dietary preference, or health status. We hope to find out the relationship between PTC sensitivity and TAS2R38 gene polymorphisms and dietary preference and health status of Chinese population through this study, providing relevant guidance and suggestions for dietary guidance and prevention of some chronic diseases in Chinese population.

**Supplementary Information:**

The online version contains supplementary material available at 10.1186/s41065-022-00260-x.

## Introduction

Bitterness is one of the most well-studied taste components. Bitter tastes are often perceived negatively and thereby serve to protect us from consuming toxic substances [1–3]. The chemical structures of substances considered bitter in everyday life are not the same, and the bitter compounds present in food include a wide range of structurally diverse molecules [4]. Bitter taste perception is a variable trait that differs from person to person [5, 6]. The most famous example of differences in bitterness sensitivity among individuals is that of phenylthiourea (PTC) and 6-n-propyl thiouracil (PROP) [1, 7]. PTC and PROP belong to the thiourea family, which contains compounds with an N–C = S (thiocyanate) group. This functional group is responsible for the bitter taste of thiourea compounds [6, 7]. However, while some humans find these compounds bitter, others are unable to perceive their bitterness [1, 8].

There are 25 distinct functional *TAS2R* genes in humans, whose products are responsible for bitter taste perception. Hence, the *TAS2R* gene family controls the differences in bitter taste perception among humans [9–11]. *TAS2R38*, the most widely studied member of this gene family, is located on chromosome 7q34. The full length of the *TAS2R38* gene is 8143 bp (DNA). The gene contains only one exon and produces mRNA that is 1143 bp in length. The human *TAS2R38* gene is associated with differences in the sensitivity to PTC bitterness and PROP [5, 12, 13]. The *TAS2R38* gene encodes a seven-transmembrane G protein-coupled receptor [9] that binds to the N–C꞊S group present in PTC and PROP [1, 6, 14]. *TAS2R38* contains three common missense single nucleotide polymorphisms (SNPs) (rs713598, rs1726866, and rs10246939). These SNPs result in the substitution of proline to alanine at amino acid position 49 (P49A), alanine to valine at position 262 (A262V), and valine to isoleucine at position 296 (V296I), respectively (Table 1) [5, 9–11, 15, 16]. These three amino acid substitutions lead to individual differences in the sensitivity to PTC bitterness and PROP [5, 11, 15, 16].

**Table 1:** SNP variations in the *TAS2R38* gene

**Variant nucleotide position**	**Variant amino acid position**	**Allele**	**Amino acid encoded**	**Predicted location**
145	49	C	Proline	First intracellular loop
		G	Alanine	
785	262	C	Alanine	Sixth transmembrane domain
		T	Valine	
886	296	G	Valine	Seventh transmembrane domain
		A	Isoleucine	

*Note*: Position of the variant nucleotide, the alternative base pairs at each variant position, position of the encoded variant amino acid, the alternative amino acids at each variant position, and the location of the variant amino acid positions are indicated in relation to the predicted secondary structure of the protein

Theoretically speaking, the three polymorphic loci of *TAS2R38* can produce eight different alleles (PAV, PAI, PVV, PVI, AAV, AAI, AVV, and AVI) in humans. These haplotypes do not occur with equal frequency, with the most common being PAV and AVI. In contrast, haplotypes such as AAV, AAI, PAI, and PVI are rare (1–5%), although they are more common in certain populations in Africa, where they are commonly found in combination with PAV or AVI and rarely in a homozygous state [16–18].

Some studies suggest that rs713598 is one of three SNPs constituting the haplotype that determines the ability to perceive the bitter taste of PTC. At the rs713598 site, carriers of the C allele, typically homozygotes, show sensitivity to PTC, while carriers of the G allele are unable to taste it [19]. Interestingly, studies have revealed that populations with the two main forms of the *TAS2R38* gene (PAV and AVI) show significant differences in the sensitivity to PTC bitterness. Population with the PAV allele can more easily perceive the bitter taste of PTC than the population with AVI. Therefore, PAV appears to represent the “taster” allele, whereas AVI represents the “non-taster” allele [5, 15, 17].

Many studies have shown that *TAS2R38* alleles affect bitter taste perception, which in turn can influence eating habits. Lipchock et al. detected the expression of *PAV-TAS2R38* in PAV/AVI heterozygotes and found that differences in *PAV-TAS2R38* expression were associated with differences in the taste of broccoli juice (higher gene expression corresponded to higher bitterness scores), but not with the bitterness rating of non-bitter solutions (i.e., sodium chloride and carrot juice). They also found a positive correlation between caffeine consumption and *PAV-TAS2R38* expression [20]. Duffy et al. found that individuals with the AVI/AVI diplotype have a significantly higher vegetable intake than individuals with other diplotypes [21]. It has also been suggested that individuals carrying the PAV/PAV diplotype are more sensitive to the bitter taste of capsaicin and ethanol than those with other diplotypes [22]. Findings by Choi et al. also indicate that the *TAS2R38* bitter taste receptor gene affects alcohol consumption behavior among humans [23].

A genetic marker study showed that variations in rs10246939, an SNP in the *TAS2R38* bitter taste receptor gene, are associated with dietary intake and the risk of obesity in populations from South Korea, and the association is more pronounced in women [24]. In contrast, in an Indian population, no significant correlation was identified between body mass index (BMI) and the presence of specific *TAS2R38* diplotypes (PAV/PAV, PAV/AVI, and AVI/AVI) [14]. Similarly, in a study from southern Italy, polymorphisms in *TAS2R38* were not found to be associated with BMI in either men or women [15]. A Japanese study found that the *TAS2R38* haplotype was associated with height and weight among college students, but not with BMI, which may influence energy and carbohydrate intake [25]. Haplotypes of the *TAS2R38* gene have also been reported to be associated with smoking status among Euro-Americans, but not in the African-American population [26].

According to existing studies, polymorphisms in *TAS2R38* and differences in PTC sensitivity have a wide impact on human taste perception, dietary preferences, lifestyle, and health; for example, TAS2R38 gene polymorphisms are well-established as underlying susceptibility to upper respiratory infection and chronic rhinosinusitis [27–29]. Although there has been substantial research on various aspects of the *TAS2R38* gene, data from Chinese populations is lacking. Therefore, the first objective of this study was to determine the frequencies of different *TAS2R38* diplotypes and the status of PTC perception among Chinese individuals. The secondary goal was to investigate the relationship of *TAS2R38* gene polymorphisms and PTC sensitivity with BMI, food preference, and overall health status in college students from China.

## Materials and methods

### Subjects

A total of 320 college students, including 133 males and 187 females, were selected from the Hubei University of Arts and Sciences. All participants were volunteers, 18–23 years of age, and were in good health at the time of sampling. The participants were briefly introduced to the study, and their informed consent was obtained. All experimental procedures and protocols were approved by the Hubei University of Arts and Science Animal Ethics Committee, China.

### Determination of genotype

The sensitivity to PTC bitterness was determined using the threshold method to identify the genotype.

#### Solution preparation

First, 1.3 g of crystallized PTC was weighed using an electronic balance and placed in a sterilized volumetric bottle. Then, 1000 mL of distilled water was added, and the mixture was shaken until completely dissolved. The resulting solution concentration was 1/750. This original solution was called the No. 1 solution. Subsequently, solutions 2–14 were prepared from solution 1 via serial dilution (Table S1).

#### Steps of testing

The PTC tasting ability of subjects was determined using the threshold method. Subjects first tasted solution No. 14; 4–5 drops of the solution were added to the base of the subjects’ tongues using a gel dropper. The subjects swallowed the solution slowly, and the same experiment was repeated with distilled water. Subjects were asked whether they could taste the difference between the two solutions. If not, the same coupled experiment was repeated with solution no. 13, solution no. 12, and so on (in decreasing order) until the subject could clearly identify the bitter taste (positive response). If a positive response was obtained, the same concentration was tested thrice. If a subject provided a positive response for all three concentrations, the results were considered reliable, and the solution number was recorded. During the experiment, subjects were asked to repeatedly taste the PTC solution and distilled water in a random order in order to exclude any bias due to speculation or any other psychological factors. If a subject could not taste bitterness even with solution no. 1, the concentration of solution no. 1 was recorded. Based on these findings, the sensitivity to PTC bitterness was graded as follows: solutions 1–6, tt (low sensitivity); solutions 7–10, Tt (medium sensitivity); and solutions 11–14, TT (high sensitivity).

### Survey

The hometown of the participants was counted. Then, according to the statistical results, the study population was divided into two groups: the Hubei Province group (Wuhan, Xiangyang, Yichang, Shiyan, Zaoyang, Jingzhou, Huangshi, Huanggang, Jingmen, Enshi, Suizhou, Xianning, Xiaogan, etc.), and the group from other parts outside Hubei province (Sichuan, Henan, Anhui, Gansu, Guangdong, Guangxi, Guizhou, Xizang, Qinghai, Shanxi, etc.).

The basic health status and daily eating habits of the subjects were investigated using a self-compiled questionnaire. The questionnaire included questions on height and weight; a history of rhinitis, gastritis, enteritis, and high blood pressure; smoking habits; preference for tea, coffee, cruciferous vegetables, oily food, meat, sweet fruits, sour fruits, cilantro, and fennel seedlings; a family history of baldness; and the perceived saltiness of foods. Participants were allowed to provide yes or no answers for each question.

### SNP typing of the *TAS2R38* gene

#### DNA extraction

DNA was extracted from oral swabs using the High Efficiency Oral Swab Genomic DNA Extraction Kit (TIANGEN Biotech [Beijing] Co., Ltd., China) based on manufacturer instructions. The DNA products were used as templates for subsequent polymerase chain reactions (PCRs).

#### Primer design

**Fig. 1:**
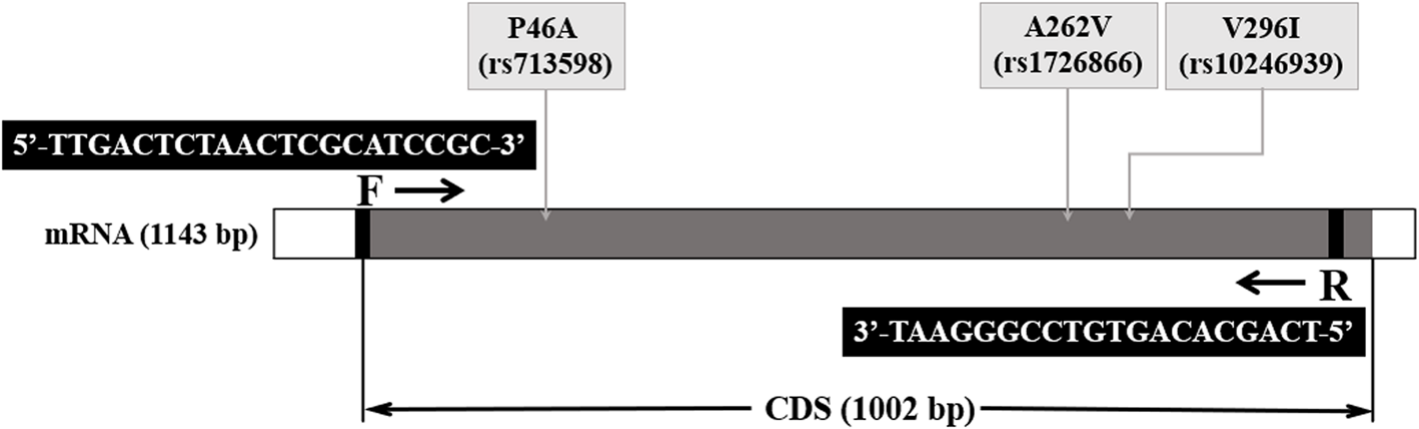
The schematic diagram of gene structure including the position of primer and SNPs

The *TAS2R38* gene sequence was downloaded from the NCBI database (https://www.ncbi.nlm.nih.gov/). The mRNA-coding site on the DNA was between nucleotides 5011 and 6143 and was 1143 bp long. Within this site, the CDS sequence was located between 85 and 1086 nucleotides (Fig. 1).

#### PCR amplification and sequencing

The PCR system was configured according to the instructions of the 2X Taq Plus Master Mix (Dye Plus) kit (Nanjing Vazyme Biotech Co., Ltd., China). The reaction volume was 50 µL, including 5 µL of diluted DNA template, 2 µL of forward and reverse primer, 25 µL of the 2X Taq Plus Master Mix (Dye Plus), and 16 µL of double-distilled water. The PCR conditions were as follows: pre-denaturation at 95℃ for 180 s; 35 cycles of denaturation at 95℃ for 30 s, annealing at 56℃ for 30 s, and extension at 72℃ for 30 s; and extension at 72℃ for 300 s. The amplified products were detected using 1% agarose gel electrophoresis. The bands were excised and mailed to Qingke Company (Wuhan, China) for 3730 sequencing.

#### Sequence comparison

Using the sequencing data, the allelic variation and haplotype frequencies were analyzed using ClustalW Multiple alignment Tool by the BioEdit Sequence Alignment Editor software. Variations were recorded and compared with the genotypes determined using the PTC taste experiment.

### Statistical analysis

Body mass index (BMI = weight [kg]/height [m]^2^) of subjects was calculated based on height and weight. IBM SPSS Statistics 21 statistical analysis software was used to analyze the data. Analysis of variance was used to analyze the relationship of *TAS2R38* genotype and the perception of PTC bitterness with BMI. Meanwhile, the Chi-square test was used to analyze the relationship of *TAS2R38* genotype and the perception of PTC bitterness with dietary preferences and lifestyle habits. The significance level was set as *P* < 0.05.

## Results

### PTC tasting ability

The PTC tasting ability of 320 subjects (133 [41.6%] male and 187 [58.4%] female; aged 18–23 years) was determined using the threshold method. The test results are shown in Table S2. Approximately 65% of individuals in the study population had a moderate sensitivity to PTC bitterness (Tt genotype), with no significant difference between male and female subjects. Among male subjects, the number of individuals with greater sensitivity to PTC bitterness (TT genotype) was significantly lower than that of individuals with low sensitivity (tt genotype). The opposite was true for women. The percentage of male subjects who were sensitive to PTC bitterness (TT genotype) was significantly lower than that of female subjects. In contrast, the proportion of male subjects who were less sensitive to PTC bitterness (tt genotype) was significantly higher than that of female subjects.

We further investigated whether the sensitivity to PTC bitterness varied among participants from different geographical regions. The geographical distribution of the study population is shown in Table S3. Approximately 65% of the study population had moderate sensitivity to PTC bitterness (Tt genotype), and there were no significant differences between individuals from different geographic regions. The proportion of subjects sensitive to PTC bitterness (TT genotype) in Hubei province was slightly higher than that in the other regions, while the proportion of subjects not sensitive to PTC bitterness (tt genotype) in Hubei Province was slightly lower than that in the other regions. In the Hubei Province population, the proportion of individuals sensitive to PTC bitterness (TT genotype) was significantly higher than that of individuals with no sensitivity to PTC bitterness (tt genotype). In subjects outside Hubei province, the proportion of individuals sensitive to PTC bitterness (TT genotype) and the proportion of individuals with no sensitivity to PTC bitterness (tt genotype) was comparable.

### *TAS2R38* polymorphisms

Table 2 shows the genotype and allele frequencies of the three *TAS2R38* SNPs in the total study population. Genotyping of the three SNPs (C145G, C785T, and G886A) yielded minor allele (C145, C785, and G886) frequencies of 0.664, 0.880, and 0.680, respectively (Table 2). Hence, the mean frequency for the study population was calculated to be 0.741.

**Table 2:** Allele frequency of *TAS2R38* SNPs in the study population

	**Genotype frequency, n (%)**				**Allele frequency**	
**rs713598**	**CC**	**CG**	**GG**	**Total**	**C**	**G**
	136 (42.5)	153 (47.8)	31 (9.7)	320 (100)	0.664	0.336
**rs1726866**	**CC**	**CT**	**TT**	**Total**	**C**	**T**
	272 (85.0)	19 (5.9)	29 (9.1)	320 (100)	0.880	0.120
**rs10246939**	**GG**	**GA**	**AA**	**Total**	**G**	**A**
	144 (45.0)	147 (45.9)	29 (9.1)	320 (100)	0.680	0.320

Each SNP G145C, T785C, and A886G corresponds to an amino acid substitution in the taste receptor variants A49P, V262A, and I296V, respectively. Haplotype analysis showed that six of the eight possible haplotypes were present in the studied population (Table 3). The PAV, AVI, and AAI haplotypes accounted for 98.13% of the observed results, while the rare haplotypes (AAV, PVV, and AVV) only accounted for 0.16–1.41% of the study population. The remaining two combinations (PVI and PAI) were not detected in the study population. Haplotype analysis revealed that the common haplotype AAI mainly appears as a heterozygous combination with PAV or AVI, but almost never appears in the homozygous form. In addition, among the common haplotypes, the frequency of the savor haplotype (PAV) was about 3.2 times that of the AAI haplotype and about 5.7 times that of the non-savor haplotype (AVI).

**Table 3:** Haplotype frequency in the study population

**Haplotype**	**Nucleotide**			**Amino acid**			**Occurrence**	**Frequency**
	**145**	**785**	**886**	**49**	**262**	**296**		
**PAV**	C	C	G	P	A	V	423	0.6609
**AVI**	G	T	A	A	V	I	74	0.1156
**AAI**	G	C	A	A	A	I	131	0.2047
**AAV**	G	C	G	A	A	V	9	0.0141
**PVV**	C	T	G	P	V	V	2	0.0031
**AVV**	G	T	G	A	V	V	1	0.0016

As shown in Table S4, among the diplotypes, the homozygous taster PAV/PAV diplotype and heterozygous PAV/AAI diplotype were the most common, with incidence rates of 42.19% and 40.63%, respectively. This was followed by the homozygous non-taster diplotype AVI/AVI (8.75%), the heterozygous diplotype PAV/AVI (5.00%), and PAV/AAV (1.88%). An analysis of diplotypes revealed that the homozygous taster (PAV/PAV) diplotype was approximately 4.8 times more common in the study population than the homozygous non-taster one (AVI/AVI).

We further investigated whether there were differences in *TAS2R38* gene polymorphisms across individuals from different geographical regions (Hubei province vs. Other regions outside Hubei province). The distribution results are shown in Table S5. Among the people in Hubei, the proportion of the homozygous taster PAV/PAV diplotype is higher than heterozygous PAV/AAI diplotype, but among people outside Hubei province, the results were reversed. The proportion of common diplotype PAV/PAV and PAV/AVI in Hubei is higher than that in other areas,but the proportion of PAV/AAI diplotype and AVI/AVI diplotype in Hubei is lower than that in other areas.

A comprehensive analysis of *TAS2R38* diplotype and PTC bitterness perception was subsequently performed (Table S6). The results showed that among individuals with moderate PTC sensitivity (genotype Tt), the PAV/AAI (46.63%) and PAV/PAV (39.90%) diplotypes were the most common. The PAV/PAV (65.67%) diplotype was the most common in individuals with high PTC sensitivity (TT), and AVI/AVI (46.67%) was the most common in individuals with no PTC sensitivity (tt).

### Relationship of *TAS2R38* genotype and sensitivity to PTC bitterness with BMI and dietary habits

No significant correlation was found between BMI among the 309 carriers of the four common diplotypes (PAV/PAV, AVI/AVI, PAV/AAI and PAV/AVI) (*P* = 0.527) (Table S7). Similarly, there was no significant association between BMI and the perception of PTC bitterness (*P* = 0.253) (Table S8).

The corresponding *P* values obtained according to the Chi-square test are shown in Table 4. For the diplotype analysis, we only included the 309 participants with common diplotypes (PAV/PAV, AVI/AVI, PAV/AAI and PAV/AVI). We found that these common diplotypes were significantly associated with tea consumption (*P* = 0.027) but not with region, gender, health status, other dietary preferences, or lifestyle (*P* > 0.05). No significant correlation was identified between PTC bitterness perception and region, health status, dietary preferences, or living habits (*P* > 0.05). However, this factor was significantly correlated with gender (*P* < 0.05). The female subjects had a better sensitivity to PTC bitterness than male subjects, and they were thus more likely to find PTC-containing foods bitter.

**Table 4:** Association of *TAS2R38* diplotypes and PTC bitterness perception with dietary preferences and health status (*P* values)

**Dietary preferences and health status**	**Common diplotypes (*n *= 309)**	**Status of PTC bitterness perception (*n*** **= 320)**
**Region (Hubei Province/Other areas)**	0.655	0.418
**Gender (Male/Female)**	0.407	0.000125
**Have or have had rhinitis (Yes/No)**	0.257	0.545
**Have a smoking habit (Yes/No)**	0.180	0.058
**Have or have had gastritis (Yes/No)**	0.872	0.695
**Have or have had enteritis (Yes/No)**	0.194	0.457
**Like to drink tea (Yes/No)**	0.027	0.542
**Like to drink coffee (Yes/No)**	0.051	0.860
**Like to eat cruciferous vegetables (Yes/No)**	0.449	0.533
**Like to eat oily food (Yes/No)**	0.595	0.197
**Like to eat meat (Yes/No)**	0.803	0.388
**Like to eat sour fruits (Yes/No)**	0.158	0.802
**Like to eat sweet fruits (Yes/No)**	0.588	0.504
**Like to eat cilantro (Yes/No)**	0.757	0.915
**Like to eat fennel shoots (Yes/No)**	0.336	0.170
**Have high blood pressure (Yes/No)**	0.350	0.395
**Have baldness in the family (Yes/No)**	0.578	0.906
**The taste is relatively salty (Yes/No)**	0.322	0.284

## Discussion

Several studies have examined the *TAS2R38* bitter taste receptor gene. However, data in the Chinese population has been lacking. To our knowledge, this study is the first to characterize the allele and haplotype frequency of *TAS2R38* in a Chinese population. Interestingly, the haplotype frequencies observed in the present study were significantly different from those reported for *TAS2R38* in previous studies. The haplotype frequency of PAV observed in the Chinese population (66.1%) in the present study is roughly consistent with that reported by Risso et al. in an Asian population (64.5%). However, the haplotype frequency of AVI observed in the present study (11.6%) is much lower than that reported by Risso et al. (35.18% in Africa, 35.31% in Asia, 49.22% in Europe, and 26.69% in the Americas). The haplotype frequency of AAI we observed in the Chinese population (20.5%) is also much higher than that reported by Risso et al. (13.22% in Africa, 0.00% in Asia, 0.55% in Europe, and 2.26% in the Americas) [30]. The frequencies of the three haplotypes PAV, AVI, and AAI, which were common in the Chinese population, were found to be 30.3%, 66.1%, and 0.0%, respectively, in the Indian population [14].

Studies have also examined the diplotype distribution of *TAS2R38*. The frequencies of PAV/PAV, PAV/AVI, and AVI/AVI were reported to be 22.5%, 44.2%, and 28.3% in a southern Italian population [15]; 35.4%, 47.5%, and 17.2% in a Korean population [23], and 9.9%, 39.7%, and 43.8% in an Indian population [14], respectively. Therefore, compared with other populations, the Chinese population had a higher proportion of PAV homozygous (42.2%), a lower proportion of AVI homozygous (8.8%), and a very low proportion of heterozygous PAV/AVI (5.0%). However, the PAV/AAI diplotype, which is not common in other populations, showed a very high frequency in the Chinese population (40.6%).

To understand the geographical distribution of common diplotypes, we divided the study population into two groups: individuals from Hubei province and individuals from outside Hubei Province. Our analysis revealed no significant difference in the distribution of common diplotypes between the two groups (*P* = 0.655).

In many studies of the *TAS2R38* gene, PAV and AVI have been found to be the most common haplotypes. Meanwhile, other haplotypes have been found to be rare (1–5%) or have only been observed frequently in certain populations [16–18]. For example, Wooding et al. found that the AAI allele, which is rare in other populations, is present in 15% of the African population [31]. Similarly, in an analysis of PTC taste in Central and West African populations, Campbell found that the frequency of the AAI haplotype was 10–20% [18]. In our study, the frequency of the AAI haplotype was as high as 20.5%, even higher than that of AVI (11.6%). However, it should be noted that in most cases, AAI appeared in a heterozygous combination with PAV (PAV/AAI). It very rarely appeared in a heterozygous combination with AVI (AVI/AAI), and did not appear in the homozygous form or with any other haplotype.

The proportion of individuals with a weak perception of PTC bitterness is known to differ across different regions. It is believed to be approximately 2.3–36.5% in Africa, 6.9–36.8% in Europe, 10% in Mexico, 15% in Korea, and 1.8–33.1% in Japan [32–34]. We divided the study population into three groups—TT (high sensitivity to PTC bitterness), Tt (medium sensitivity), and tt (no sensitivity)—according to the perception of PTC bitterness. We found that 14.06% of our study population had a low sensitivity to PTC bitterness (tt genotype), which was largely comparable to reported data (5.1–23%) [35]. We also analyzed the geographical distribution of PTC perception and found no significant difference in the perception of PTC bitterness among people from within and outside Hubei Province (*P* = 0.418).

Several studies have shown that individuals sensitive to PTC bitterness have one or two dominant alleles (PAV/PAV or PAV/AVI), while those who cannot taste PTC bitterness have recessive homozygous genes (AVI/AVI) [10, 17, 36]. Our results revealed a strong correlation between the *TAS2R38* diplotype and PTC taste perception (*P* < 0.01) in the study population. In our study, 65.67% of individuals who were sensitive to PTC bitterness (TT genotype) had a PAV/PAV diplotype. Meanwhile, 46.67% of individuals who were not sensitive to PTC bitterness (tt genotype) had an AVI/AVI diplotype. These findings were consistent with the reported results.

In our study population, some individuals who were highly sensitive to PTC bitterness (TT genotype) had an AVI/AVI diplotype, and some with low sensitivity had a PAV/PAV diplotype. This discrepancy can be explained by the findings of Behren et al., who suggested that although the sensitivity to PTC bitterness and related compounds is largely driven by a simple “taste” (PAV) and “non-taste” (AVI) dichotomy, genetic diversity could result in a large number of functional variants. Moreover, a series of “intermediate taste” alleles have also been identified, suggesting that bitterness perception for substances such as PTC is actually a complex trait [37]. Studies by Boxer et al. also indicate individuals have a wide range of sensitivity to the bitter taste of PTC and related compounds, and people may not exclusively be non-tasters, medium tasters, or supertasters [38]. Interestingly, Hayes et al. also found that the bitterness of PTC and related compounds cannot entirely be explained by the *TAS2R38* genotype, as individuals with PAV/PAV were not always sensitive to the bitterness of these compounds and those with AVI/AVI and a high number of fungiform papillae may also be sensitive to the bitterness. They suggested that one or two copies of the PAV allele were sufficient to increase the detection threshold from non-taster to taster [39]. Campbell et al.’s genetic analysis of sensitivity to PTC bitterness showed that common and rare variants can work together to significantly influence normal phenotypic variations, suggesting that alleles other than PAV and AVI could also contribute independently and differently to the observed phenotype [18]. Melis et al. proposed that chemicals in saliva could also affect the perception of bitter compounds such as PTC [40]. Further, some researchers also believe that other factors that affect taste, such as aging and oral diseases, may also affect the sensitivity to PTC bitterness. However, the current evidence is limited, and more studies are required to fully elucidate this phenomenon [15, 41, 42].

Bartoshuk et al. reported gender differences in the perception of PTC bitterness, finding that women were more likely to perceive the bitterness of compounds such as PTC. They suggested that this was because of anatomical differences because women have more fungous papillae and taste buds [43]. Our results also revealed a significant correlation between the sensitivity to PTC bitterness and gender (*P* < 0.05), with female subjects showing a higher sensitivity than their male counterparts. However, analyses of gender differences in the common *TAS2R38* diplotypes revealed no differences between men and women (*P* = 0.407).

We analyzed the correlation between BMI and sensitivity to PTC bitterness among the study participants but found no correlation between the two (*P* = 0.253). This was consistent with existing studies [44–46]. We also analyzed the correlation between *TAS2R38* diplotypes and BMI and found a lack of direct correlation (*P* = 0.527), consistent with the findings of Sausenthaler et al. [47].

Many researchers have examined the relationship of sensitivity to PTC bitterness and *TAS2R38* gene polymorphisms with health and dietary preferences among the study population. Bell et al. found that the ability to perceive the bitter taste of PTC affects whether children enjoy eating vegetables [48]. In line with this, Negri et al. also found that children who are more sensitive to the bitterness of PTC compounds have a lower preference for vegetables [49]. Cont et al. suggested that the differences in the *TAS2R38* gene are associated with complementary feeding behavior in infants [50]. Mikołajczyk-Stecyna et al. suggested that *TAS2R38* gene polymorphisms may influence the consumption of coffee and white cabbage, but not that of other bitter foods, in older women [51]. The study by Choi et al. found that *TAS2R38* may determine the risk of gastric cancer in Korean individuals, but that *TAS2R38* diplotypes do not affect dietary intake and food, alcohol, or cigarette consumption in this population [52]. O’Brien et al. also found that the sensitivity to PTC bitterness and the *TAS2R38* genotypes that affect this sensitivity do not have a significant impact on dietary intake [53]. In addition, a large number of studies have associated the *TAS2R38* genotype with upper respiratory tract infection susceptibility, and identified *TAS2R38* single nucleotide polymorphisms associated with the course of chronic rhinosinusitis [27–29, 54]. Our study assessed whether the participants were smokers; had a history of rhinitis, gastritis, enteritis, or blood pressure; loved to drink tea or coffee or consume cruciferous vegetables, oily foods, meat, sweet fruits, sour fruits, coriander, or fennel; had a family history of baldness; or had a high sensitivity to salty tastes. Statistical analysis revealed no significant correlation between the perception of PTC bitterness and these factors (*P* > 0.05). However, polymorphisms in the *TAS2R38* gene were associated with a preference for tea (*P* = 0.027), although they showed no association with an individual’s native place, gender, health status, or other dietary habits (*P* > 0.05).

Food preferences are not influenced by a single factor. Polymorphisms in the *TAS2R38* gene can affect the sensitivity to bitterness in food and thus affect dietary preferences. However, *TAS2R38* is only one of many unique human bitter taste genes (*TAS2Rs*), and other bitter taste genes can also contribute to dietary preferences [5, 55]. In addition to genetics, food preferences can be influenced by several factors, such as a person’s own sensilla, differences between individuals, acculturation, and perceived health benefits [55, 56]. Our study only considered the effect of polymorphisms of TAS2R38 gene on dietary preference, which may affect our final results.

In addition, for the investigation and analysis of health status, the research group we choose is young college students aged 18–23, whose health status itself is at a high level, which may also affect the results of our analysis. Finally, the relatively small study population may also limit the potential to explore these relationships.

## Conclusion

We examined the polymorphisms in the *TAS2R38* bitter taste receptor gene among 320 Chinese college students and determined their sensitivity to the bitter taste of PTC. We then analyzed the association of *TAS2R38* polymorphisms and bitter taste sensitivity to PTC with an individual’s native place, gender, BMI, dietary preferences, and health status. The results showed that the PAV/AAI diplotype, which is not common in other populations, accounts for a very high proportion of the Chinese population. The haplotype AAI was more commonly detected in our study than in other populations, and was even more common than AVI. In most cases, AAI appeared in the heterozygous form along with PAV (PAV/AAI), although in some cases it appeared in the heterozygous form along with AVI (AVI/AAI). However, it was not detected in the homozygous form or in other heterozygous forms. Geography-based analyses revealed no significant correlation between *TAS2R38* polymorphisms and sensitivity PTC bitterness and an individual’s native place (within vs. outside Hubei province). Studies on gender differences revealed that the perception of PTC bitterness was higher among female subjects than among their male counterparts, although there was no correlation between the common diplotypes of the *TAS2R38* gene and gender. Further analyses revealed that *TAS2R38* gene polymorphisms were associated with an individual’s preference for tea, but not with BMI, health status, or other dietary preferences. No significant correlation was found between the perception of PTC bitterness and BMI, dietary preferences, and health status in our study population. Finally, our study showed that the perception of PTC bitterness and *TAS2R38* gene polymorphisms were not significantly correlated with dietary preference and health status of Hubei college students. However, it is important to note that there is no current study in Chinese population that has linked the perception of PTC bitterness and *TAS2R38* genotype with dietary preference and health status, which increases the necessity of research in this field and may provide relevant guidance and suggestions for dietary guidance and prevention of some chronic diseases in Chinese population.

## Supplementary Information

**Additional file 1: Table S1.** Preparation method for the PTC solutions used for genotype determination. **Table S2.** PTC tasting ability in the studied population. **Table S3.** Genotypic distribution of PTC tasting ability in different regions. **Table S4.** Diplotypic distribution of the TAS2R38 gene in the study population. **Table S5.** Diplotypic distribution of the TAS2R38 gene in different geographic regions. **Table S6.** TAS2R38 diplotypes and perception of PTC bitterness in the study population. **Table S7.** BMI according to TAS2R38 status. **Table S8.** BMI according to PTC status.

## Data Availability

The data that support the findings of this study are available from the corresponding author upon reasonable request.

## Abbreviations

PTC: phenylthiourea
BMI: body mass index
PCR: polymerase chain reaction
PROP: 6-n-propyl thiouracil
DNA: Deoxyribonucleic Acid
mRNA: messenger Ribonucleic Acid
SNP: single nucleotide polymorphism
P: Proline
A: Alanine
V: Valine
I: Isoleucine
NCBI: National Coalition Building Institute
CDS: Coding sequence
