# Supplementary material for: Variations in the TAS2R38 gene among college students in Hubei
Source: Hereditas. 2022 Dec 19;159:46. doi: 10.1186/s41065-022-00260-x (PMC9762079; doi:10.1186/s41065-022-00260-x)
Supplement: Supplementary file 1 — Additional file 1: Table S1. Preparation method for the PTC solutions used for genotype determination. Table S2. PTC tasting ability in the studied population. Table S3. Genotypic distribution of PTC tasting ability in different regions. Table S4. Diplotypic distribution of the TAS2R38 gene in the study population. Table S5. Diplotypic distribution of the TAS2R38 gene in different geographic regions. Table S6. TAS2R38 diplotypes and perception of PTC bitterness in the study population. Table S7. BMI according to TAS2R38 status. Table S8. BMI according to PTC status. [file 41065_2022_260_MOESM1_ESM.docx]

**Table S1 Preparation method for the PTC solutions used for genotype determination**

| **Number** | **Preparation Methods** | **Concentration** | | **Tasting Ability/Genotype** | |
| --- | --- | --- | --- | --- | --- |
| No. 1 | 1.3 g of PTC+ 1000 mL of distilled water | 1/750 | tt | |  |
| No. 2 | 100 mL of No. 1 + 100 mL of distilled water | 1/1500 | tt | |  |
| No. 3 | 100 mL of No. 2 + 100 mL of distilled water | 1/3000 | tt | |  |
| No. 4 | 100 mL of No. 3 + 100 mL of distilled water | 1/6000 | tt | |  |
| No. 5 | 100 mL of No. 4 + 100 mL of distilled water | 1/12000 | tt | |  |
| No. 6 | 100 mL of No. 5 + 100 mL of distilled water | 1/24000 | tt | |  |
| No. 7 | 100 mL of No. 6 + 100 mL of distilled water | 1/48000 | Tt | |  |
| No. 8 | 100 mL of No. 7 + 100 mL of distilled water | 1/96000 | Tt | |  |
| No. 9 | 100 mL of No. 8 + 100 mL of distilled water | 1/192000 | Tt | |  |
| No. 10 | 100 mL of No. 9 + 100 mL of distilled water | 1/384000 | Tt | |  |
| No. 11 | 100 mL of No. 10 + 100 mL of distilled water | 1/768000 | TT | |  |
| No. 12 | 100 mL of No. 11 + 100 mL of distilled water | 1/1536000 | TT | |  |
| No. 13 | 100 mL of No. 12 + 100 mL of distilled water | 1/3072000 | TT | |  |
| No. 14 | 100 mL of No. 13 + 100 mL of distilled water | 1/5144000 | TT | |  |

**Table S 2 PTC tasting ability in the studied population**

| **Genotype** | **Men (n=133)** | **Women (n=187)** | **Total (n=320)** |
| --- | --- | --- | --- |
| **TT** | 18 (13.53%) | 49 (26.20%) | 67 (20.94%) |
| **Tt** | 85 (63.91%) | 123 (65.78%) | 208 (65.00%) |
| **tt** | 30 (22.56%) | 15 (8.02%) | 45 (14.06%) |

**Table S 3 Genotypic distribution of PTC tasting ability in different regions**

| **Genotype** | **Hubei Province**  **(n=207)** | **Other Regions**  **(n=113)** | **Total**  **(n=320)** |
| --- | --- | --- | --- |
| **TT** | 47 (22.71%) | 20 (17.70%) | 67 (20.94%) |
| **Tt** | 134 (64.73%) | 74 (65.49%) | 208 (65.00%) |
| **tt** | 26 (12.56%) | 19 (16.81%) | 45 (14.06%) |

**Table S 4 Diplotypic distribution of the TAS2R38 gene in the study population**

|  | **Men (n=133)** | **Women (n=187)** | **Total (n=320)** |
| --- | --- | --- | --- |
| **PAV/PAV** | 56 (42.11%) | 79 (42.25%) | 135 (42.19%) |
| **AVI/AVI** | 15 (11.28%) | 13 (6.95%) | 28 (8.75%) |
| **PVV/PVV** | 1(0.75%) | 0 (0.00%) | 1 (0.31%) |
| **AAV/AAV** | 1(0.75%) | 0 (0.00%) | 1 (0.31%) |
| **PAV/AVI** | 8 (6.02%) | 8 (4.28%) | 16 (5.00%) |
| **PAV/AAV** | 3 (2.26%) | 3 (1.60%) | 6 (1.88%) |
| **PAV/AAI** | 49 (36.84%) | 81 (43.32%) | 130 (40.63%) |
| **PAV/AVV** | 0 (0.00%) | 1 (0.53%) | 1 (0.31%) |
| **AVI/AAI** | 0 (0.00%) | 1 (0.53%) | 1 (0.31%) |
| **AVI/AAV** | 0 (0.00%) | 1 (0.53%) | 1 (0.31%) |

**Table S 5 Diplotypic distribution of the TAS2R38 gene in different geographic regions**

|  | **Hubei Province (n=207)** | **Other Regions (n=113)** | **Total**  **(n=320)** |
| --- | --- | --- | --- |
| **PAV/PAV** | 92 (44.44%) | 43 (38.05%) | 135 (42.19%) |
| **AVI/AVI** | 16 (7.73%) | 12 (10.62%) | 28 (8.75%) |
| **PVV/PVV** | 1 (0.48%) | 0 (0.00%) | 1 (0.31%) |
| **AAV/AAV** | 0 (0.00%) | 1 (0.88%) | 1 (0.31%) |
| **PAV/AVI** | 11 (5.31%) | 5 (4.42%) | 16 (5.00%) |
| **PAV/AAV** | 3 (1.45%) | 3 (2.65%) | 6 (1.88%) |
| **PAV/AAI** | 82 (39.61%) | 48 (42.48%) | 130 (40.63%) |
| **PAV/AVV** | 0 (0.00%) | 1 (0.88%) | 1 (0.31%) |
| **AVI/AAI** | 1 (0.48%) | 0 (0.00%) | 1 (0.31%) |
| **AVI/AAV** | 1 (0.48%) | 0 (0.00%) | 1 (0.31%) |

**Table S 6 TAS2R38 diplotypes and perception of PTC bitterness in the study population**

|  | **TT (67)** | **Tt (208)** | **tt (45)** | **Total (320)** |
| --- | --- | --- | --- | --- |
| **PAV/PAV** | 44 (65.67%) | 83 (39.90%) | 8 (17.78%) | 135 (42.19%) |
| **AVI/AVI** | 1 (1.49%) | 6 (2.88%) | 21 (46.67%) | 28 (8.75%) |
| **PVV/PVV** | 0 (0.00%) | 1 (0.48%) | 0 (0.00%) | 1 (0.31%) |
| **AAV/AAV** | 0 (0.00%) | 1 (0.48%) | 0 (0.00%) | 1 (0.31%) |
| **PAV/AVI** | 1 (1.49%) | 13 (6.25%) | 2 (4.44%) | 16 (5.00%) |
| **PAV/AAV** | 0 (0.00%) | 6 (2.88%) | 0 (0.00%) | 6 (1.88%) |
| **PAV/AAI** | 21 (31.34%) | 97 (46.63%) | 12 (26.67%) | 130 (40.63%) |
| **PAV/AVV** | 0 (0.00%) | 1 (0.48%) | 0 (0.00%) | 1 (0.31%) |
| **AVI/AAI** | 0 (0.00%) | 0 (0.00%) | 1 (2.22%) | 1 (0.31%) |
| **AVI/AAV** | 0 (0.00%) | 1 (0.48%) | 0 (0.00%) | 1 (0.31%) |

**Table S 7 BMI according to TAS2R38 status**

|  | **Number** | **Mean value** | **Standard deviation** | **Standard error** | **95% confidence interval of the mean** | | **Minimum** | **Maximum** |
| --- | --- | --- | --- | --- | --- | --- | --- | --- |
|  |  |  |  |  | **Minimum** | **Maximum** |  |  |
| **PAV/PAV** | 135 | 20.82 | 2.99 | 0.26 | 20.31 | 21.33 | 16.22 | 30.45 |
| **AVI/AVI** | 28 | 20.46 | 1.73 | 0.33 | 19.78 | 21.13 | 16.69 | 23.46 |
| **PAV/AAI** | 130 | 20.95 | 2.95 | 0.26 | 20.44 | 21.46 | 16.41 | 31.25 |
| **PAV/AVI** | 16 | 21.75 | 2.76 | 0.69 | 20.28 | 23.23 | 17.48 | 26.73 |
| **Total** | 309 | 20.89 | 2.87 | 0.16 | 20.57 | 21.21 | 16.22 | 31.25 |

**Table S 8 BMI according to PTC status**

|  | **Number** | **Mean value** | **Standard deviation** | **Standard error** | **95% confidence interval of the mean** | | **Minimum** | **Maximum** |
| --- | --- | --- | --- | --- | --- | --- | --- | --- |
|  |  |  |  |  | **Minimum** | **Maximum** |  |  |
| **TT** | 67 | 20.63 | 2.86 | 0.35 | 19.94 | 21.33 | 16.23 | 29.21 |
| **Tt** | 208 | 21.09 | 3.00 | 0.21 | 20.68 | 21.50 | 16.22 | 31.25 |
| **tt** | 45 | 20.43 | 1.89 | 0.28 | 19.86 | 20.99 | 17.32 | 24.69 |
| **Total** | 320 | 20.90 | 2.84 | 0.16 | 20.59 | 21.21 | 16.22 | 31.25 |
